# Green's Encyclopedia Medica

**Published:** 1915-06

**Authors:** 


**Green's Encyclopedia Medica.** Second Edition. Vol. I—Abbatoirs to Asphyxia. Pp. xv. 744. Edinburgh and London: Wm. Green & Sons. 1915. Price 20s. net.

This combination of a medical encyclopedia with the medical dictionary was published some fifteen years ago. We then remarked, vol. xxiv., p. 363, that *Green's Dictionary* would be looked upon as a necessary part of the furniture of the consulting room. The general plan of the work is still maintained, the editor being responsible for the dictionarial part of the work, whilst the longer articles are written by the most eminent specialists in the British Islands. In this, the first volume of the second edition, a complete revision has been made, and every article has been revised by its author or some competent authority. It was found to be impossible to adopt the Basle Anatomical Nomenclature on this occasion, as the terminology of Basle would have been inconvenient both to writers and readers.

The illustrations have been increased in number and improved in quality, whilst the type is excellent and the printing admirable.

The special original articles amount to forty-eight in number, eight of them being under the general heading "Abdomen." These occupy nearly the first hundred pages, and about one-seventh of the whole volume. The names of the authors are a sufficient guarantee of the quality of their work.

Under the heading of "Anatomy" the Basle Anatomical Nomenclature (B.N.A.) is fully detailed in a new article of nearly one hundred pages by Professor Jamieson, of Edinburgh.

A detailed review of a work of this kind is impossible and undesirable; it must speak for itself, and will do so efficiently when consulted by an inquirer after the latest knowledge on the particular subject under the first letter of the alphabet.

